# SILS: Is It Cost- and Time-Effective Compared to Standard Pediatric Laparoscopic Surgery?

**DOI:** 10.1155/2012/807609

**Published:** 2012-07-30

**Authors:** Saidul Islam, Stephen D. Adams, Anies A. Mahomed

**Affiliations:** Department of Pediatric Surgery, Royal Alexandra Children's Hospital, Brighton BN2 5BE, UK

## Abstract

The aim of the study was to review our experience with single-incision laparoscopic surgery (SILS) and to compare costs and operative time to standard laparoscopic surgery (SLS). A prospectively collected database of operative times and costs was analysed for the years 2008–2011. SILS cases were compared to standard laparoscopy on a procedure-matched basis. Patient demographics, on-table time and consumable costs were collated. Descriptive statistics and Mann-Whitney *U*-test were utilized with SPSS for windows. Analysis of the data demonstrate that neither consumable costs nor operative time were significantly different in each group. Comparing operative costs, SILS appendicectomy, nephrectomy/heminephrectomy, and ovarian cystectomy/oophorectomy showed cost benefit over SLS (**£**397 versus **£**467; **£**942 versus **£**1127; **£**394 versus **£**495). A trend toward higher cost for SILS Palomo procedure is noted (**£**734 versus **£**400). Operative time for SILS appendicectomy, nephrectomy/heminephrectomy, and Palomo was lower compared to SLS (60 versus 103 minutes[mins.]; 130 versus 60 mins.; 60 versus 80 mins.). In conclusion, SILS appears to be cost-effective for the common pediatric surgical operations. There is no significant difference in operating time in this series, but small sample size is a limiting factor. Studies with larger numbers will be necessary to validate these initial observations.

## 1. Introduction

SILS is increasingly being used by pediatric surgeons to perform common abdominal and urological procedures. Appendectomy, cholecystectomy, nephrectomy, hysterectomy, oophorectomy, adrenalectomy, gastric bypass, Nissen fundoplication, hernia repair, splenectomy, and colon resection are some of the procedures which are routinely undertaken by the SILS approach in some centres [1–5]. The proponents of SILS over SLS give reasons of cost efficiency (avoiding use of additional ports), cosmesis (due to fewer port site incisions), less post-operative pain and earlier recovery from surgery [6]. However, the major drawbacks reported for SILS have longer operative time due to a learning curve and reduced triangulation of instruments translating into poor ergonomics particularly for the longer procedures. Moreover, there is a paucity of information on the real cost of SILS surgery. The intention of this study in comparing costs and operative time of SILS versus SLS was to improve data to enable effective health care fiscal planning.

## 2. Material and Methods

A prospectively collected database of operative times and costs was analysed for the years 2008–2011. The cost of 18 consumable items utilized during the procedures were documented and analysed. SILS cases were compared to SLS on a procedure matched basis. The number of cases in the two groups was (*n* = 21) versus (*n* = 72), respectively. The operations that were compared included appendicectomy, nephrectomy/heminephrectomy, ovarian cystectomy/oophorectomy, and Palomo procedure. 

Patient demographics, on-table time, and consumable costs were collated. Consumables for SILS included use of either Olympus TriPort (Advanced Surgical Concepts, Bray, Ireland) or Covidien SILS port (Covidien, Dublin, Ireland). In order to curtail costs, standard straight laparoscopic instruments were utilized during SILS operations. Descriptive statistics and Mann-Whitney *U*-test were utilized with SPSS for windows. Data is quoted as median (range), and *P* < 0.05 was regarded as significant.

## 3. Result

A total of 93 cases were analysed in the study: SILS (*n* = 21) and SLS (*n* = 72). 

For appendicectomy, SILS was performed in ten patients (*n* = 10) and SLS in forty eight patients (*n* = 48). The median cost for SILS was *£*397 (280–603) and in SLS was *£*467 (175–758), *P* = 0.64. Operative time in SILS was 60 minutes (60–140) and in SLS was 103 min (40–215), *P* = 0.10.

For nephrectomy/heminephrectomy, four patients (*n* = 4) had a SILS procedure with nine patients (*n* = 9) subjected to SLS. The median cost in SILS was *£*942 (779–974) and in SLS was *£*1127 (520–1595), *P* = 0.11. Operative time in SILS was 130 minutes (90–180) and in SLS was 160 minutes (70–235), *P* = 0.21.

For ovarian cystectomy/oophorectomy, SILS was conducted in four patients (*n* = 4) and SLS in six patients (*n* = 6). The median cost in SILS was *£*394 (223–702), whereas in SLS was *£*495 (246–729), *P* = 0.56. Operative time in SILS was 90 minutes (60–120) and in SLS was 80 minutes (60–130), *P* = 0.10.

For Palomo varicocele procedure, SILS was performed in three patients (*n* = 3), SLS in nine patients (*n* = 9). The median cost in SILS was *£*734 (532–735) and in SLS was *£*400 (205–801), *P* = 0.07. Operative time in SILS was 60 minutes (50–60) and in SLS was 80 minutes (55–100), *P* = 0.17.

Comparison of operating costs in SILS and SLS is shown in Table 1. Comparison of operative time in SILS and SLS is shown in Table 2.

## 4. Discussion

Recent publications have established the feasibility of SILS in the pediatric population [1–5]. However to become a truly established method of performing surgery in children, SILS has to be demonstrated to be economically feasible. We attempted to achieve this by prospectively documenting the consumable cost, times of operation, and demographic data for all laparoscopic procedures and undertaking a comparative assessment of cost and operating time between SILS and SLS for common pediatric surgery operations.

Apart from Palomo procedure where costs were higher, SILS was found to be more cost-effective than SLS in appendicectomy, nephrectomy/heminephrectomy, and ovarian cystectomy/oophorectomy. However, this did not translate into statistical significance because of the small sample size. The higher cost of SLS was largely due to the use of additional port/ports which were more expensive relative to the cost of the SILS port. Once access into the abdomen was achieved, instrument and haemostatic devices use was broadly similar. The higher cost of Palomo was a surprise given the simplicity of the procedure, and this is attributed to the inadvertent opening of an ultrasonic haemostatic device in addition to a hemoclip for a single case when just the latter would have sufficed. Given the small number of patients this additional cost for the SILS group was sufficient to adversely influence the figures.

Operative time in SILS was lower than SLS for appendicectomy, nephrectomy/heminephrectomy, and Palomo procedure. This is due possibly to the fact that all SILS procedures were performed by a single laparoscopic surgeon with extensive experience. A prospective randomized trial from the adult literature has shown that duration of operation is significantly shorter with traditional laparoscopy compared to SILS [7]. Currently, prospective randomized trial in children are in progress assessing operative time as a primary outcome variable in these two groups of patients.

Initially Olympus TriPort ports were utilized, but this was changed to Covidien SILS port for two reasons: firstly, standardization of consumable procurement within the department and secondly, an improved gas seal provide by the latter. This change was cost-neutral. Generally costs for the SILS approach were contained by the use of standard, reusable laparoscopic instruments [8]. Instrument clash is a challenge with straight devices, but the difficulties are not insurmountable and all operations were completed without conversion [9]. Clearly roticulating and curved instruments or even magnetic graspers while improving ergonomics and maneuverability when used with SILS, require practice to master and involve significant additional costs [9, 10].

Hansen et al. [11], in his series of 224 SILS in children, reported at least 21% of operations requiring one additional port even for commonly performed operations like appendicectomy. In our series, none of the SILS cases required additional ports.

Although not the subject of this study, it is possible that there are other indirect cost benefits of the SILS approach, namely, an improved pain experience and thus analgesic usage from the use of fewer ports and cost savings from earlier hospital discharge. Additionally, there are clear benefits to the national economy from earlier return to work.

In conclusion, SILS appears to be cost-effective and safe for common pediatric surgical operations. There are no significant differences in operating time compared to standard laparoscopy in this series, but we are limited by a small sample size. Studies with larger numbers will be necessary to validate these initial observations.

## Figures and Tables

**Table 1:** Comparison of operative costs, median (range).

Total (*n* = 93)	SILS (*n* = 21)	Laparoscopy (*n* = 72)	*P* value
Appendicectomy cost (GBP)	*n* = 10 397 (280–603)	*n* = 48 467 (175–758)	0.64
Nephrectomy/heminephrectomy cost (GBP)	*n* = 4 942 (779–974)	*n* = 9 1127 (520–1595)	0.11
Ovarian cystectomy/oophorectomy cost (GBP)	*n* = 4 394 (223–702)	*n* = 6 495 (246–729)	0.56
Palomo cost (GBP)	*n* = 3 734 (532–735)	*n* = 9 400 (205–801)	0.07

**Table 2:** Comparison of operative time, median (range).

Operative time	SILS (mins.) median (range)	Laparoscopy (mins.) median (range)	*P* value
Appendicectomy	60 (60–140)	103 (40–215)	0.10
Nephrectomy/heminephrectomy	130 (90–180)	160 (70–235)	0.21
Ovarian cystectomy/oophorectomy	90 (60–120)	80 (60–130)	0.10
Palomo	60 (50–60)	80 (55–100)	0.17
